# SUSTain: Scalable Unsupervised Scoring for Tensors and its Application to Phenotyping

**DOI:** 10.1145/3219819.3219999

**Published:** 2018-07

**Authors:** Ioakeim Perros, Evangelos E. Papalexakis, Haesun Park, Richard Vuduc, Xiaowei Yan, Christopher Defilippi, Walter F. Stewart, Jimeng Sun

**Affiliations:** Georgia Institute of Technology; University of California, Riverside; Georgia Institute of Technology; Georgia Institute of Technology; Sutter Health; Inova Heart and Vascular Institute; Sutter Health; Georgia Institute of Technology

**Keywords:** Tensor Factorization, Matrix Factorization, Phenotyping, Unsupervised learning

## Abstract

This paper presents a new method, which we call SUSTain, that extends real-valued matrix and tensor factorizations to data where values are integers. Such data are common when the values correspond to event counts or ordinal measures. The conventional approach is to treat integer data as real, and then apply real-valued factorizations. However, doing so fails to preserve important characteristics of the original data, thereby making it hard to interpret the results. Instead, our approach extracts factor values from integer datasets as *scores* that are constrained to take values from a small integer set. These scores are easy to interpret: a score of zero indicates no feature contribution and higher scores indicate *distinct levels* of feature importance.

At its core, SUSTain relies on: a) a problem partitioning into integer-constrained subproblems, so that they can be optimally solved in an efficient manner; and b) organizing the order of the subproblems’ solution, to promote reuse of shared intermediate results. We propose two variants, SUSTain_*M*_ and SUSTain_*T*_, to handle both matrix and tensor inputs, respectively. We evaluate SUSTain against several state-of-the-art baselines on both synthetic and real Electronic Health Record (EHR) datasets. Comparing to those baselines, SUSTain shows either significantly better fit or orders of magnitude speedups that achieve a comparable fit (up to 425× faster). We apply SUSTain to EHR datasets to extract patient phenotypes (i.e., clinically meaningful patient clusters). Furthermore, 87% of them were validated as clinically meaningful phenotypes related to heart failure by a cardiologist.

## 1 INTRODUCTION

Matrix and tensor factorization are among the most promising approaches to extracting meaningful latent structure from multi-aspect data. They have been applied successfully in diverse applications, including social network analysis [31], text mining [14], image processing [35], recommendation systems [26], brain data analysis [2] and healthcare analytics [22], to name a few. Factorization models decompose input data into real-valued representatives revealing clusters with distinct interpretable feature profiles.

However, a significant problem arises when the input data are most naturally expressed as integer values. Examples include event counts and ordinal data [15]. In such cases, real-valued factors distort the original integer characteristics. For example, real values might no longer be interpretable as counts or frequencies. Also, the possible ranges and relative differences of elements in real-valued factors is arbitrary; this makes it hard to intuitively compare the importance of different elements. Furthermore, in many applications, practitioners are accustomed to interpreting integer-valued scores in standardized scales. Real-valued factors might require arbitrary thresholding or other unnatural transformations to convert into such scales, thereby inhibiting interpretation by domain experts.

A specific motivating application for our methods is clinical phenotyping from Electronic Health Records (EHR) data. Consider that a disease, such as heart failure, is often heterogeneous in that patients differ by underlying pathophysiology and needs. That is, a disease is often comprised of distinct disease subtypes, or phenotypes, which vary by the ensemble of causes, associations with other diseases, and treatment needs [52]. Phenotyping is intended to distinguish the latent structure among features that can, in turn, be used to prevent disease subtypes and improve treatment development and management [43]. EHR data offer a diverse and rich set of features (e.g., diagnostic, drug and procedure codes) that can serve to improve disease phenotyping. But, these data must often be represented in integer form (e.g., clinical event counts) to be utilized in unsupervised learning. For example, we can construct a patient-disease matrix where the *ij*-th element represents the number of times patient *i* had disease *j* documented in her records. Similarly, we can build higher-order tensors such as a patient-disease-medication one. The goal of unsupervised phenotyping is to identify patient clusters defined by unique feature sets, each one of which aligns with a distinct and intuitive clinical profile; in this work, we tackle this challenge via a scalable constrained integer tensor factorization.

Factorization methods have been successfully used for EHR-based unsupervised phenotyping [21, 22, 24, 41, 42, 50]. In many of those settings, the problem can be formulated via Nonnegative Matrix Factorization (NMF) [35] e.g., minimizing the squared Frobenius norm of the error:

(1) $$\text{min}\left\{{\left\|X-U\;V^{T}\right\|}_{F}^{2}|U\geq 0,V\geq 0\right\}$$

$X\in {\mathbb{Z}}_{+}^{M\times N}$ is a non-negative integer input matrix whose ***X***(*i*, *j*) cell reflects the event counts for the *i*-th (out of *M*) patient with respect to the *j*-th (out of *N*) features. Given an input number *R* of desired phenotypes, the matrix $U\in {\mathbb{R}}^{M\times R}$ corresponds to a membership matrix of the patients with respect to the *R* phenotypes. And the matrix $V\in {\mathbb{R}}^{N\times R}$ provides the phenotypes’ definition: the non-zero elements of the *r*-th column ***V*** (:, *r*) reveal the potentially relevant features to the *r*-th phenotype.

Interpreting those factors is crucial in order to determine whether and to what extent a patient exhibits a phenotype, as well as which set of candidate features should be considered to compose each *r*-th phenotype so that it is clinically meaningful. However, this can be challenging if the resulting factors contain arbitrary (non-negative) real values. Real-valued factors distort the count nature of input data; thus, identifying cases and controls based on counts of relevant medical features [13, 34] is no longer possible. Also, the possible ranges and relative differences of elements in real-valued factors is arbitrary, thus impeding the practitioner’s assessment of their relative importance. In practice, ad hoc heuristics have been introduced with limited success: a) hard thresholding to the ranked list of factor elements, which is usually arbitrary and leads to poor model fit; b) the factor values are hidden altogether and only the elements’ ranking is preserved, which omits valuable information regarding the individual elements’ actual importance.

### Contributions:

To tackle these challenges, we propose Scalable Unsupervised Scoring for Tensors (SUSTain), a framework extracting the factor values as *scores*, constrained to a small integer set. SUSTain offers a straightforward interpretation protocol: a score of zero indicates no feature contribution and higher scores indicate *distinct levels* of feature importance.

Our methodology relies on identifying a problem partitioning into integer-constrained sub-problems so that each one of them can be solved optimally in an efficient manner; at the same time, their solution order is organized so as to promote re-use of shared intermediate results. SUSTain can handle both matrix and tensor inputs, through SUSTain_*M*_ and SUSTain_*T*_ methods, which we formulate in Sections 3.1 and 3.2 respectively.

**Figure 1:**
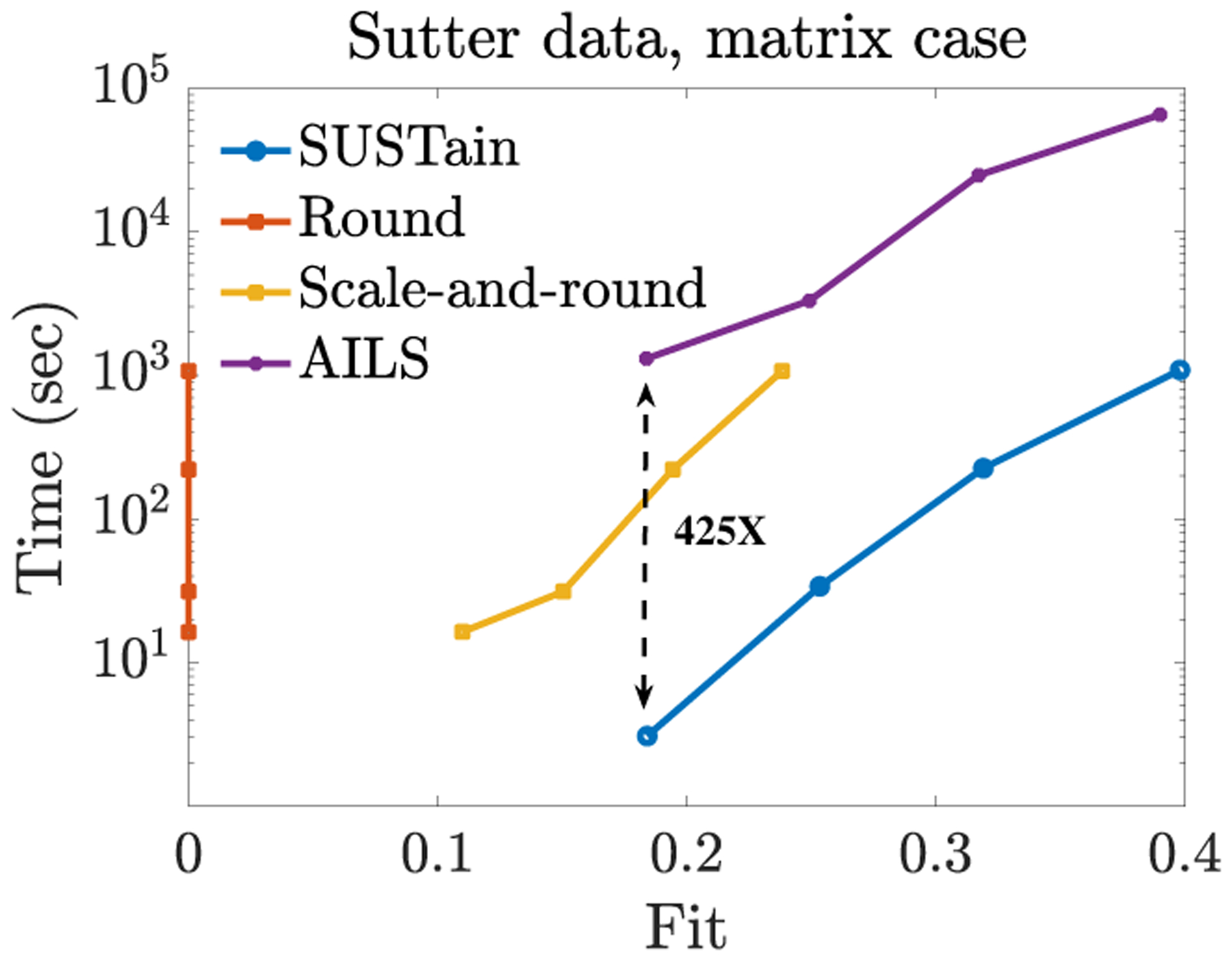
Fit (range [0, 1]) vs time trade-off for varying target number of phenotypes *R* = {5, 10, 20, 40}, on a patients-by-diagnoses matrix formed of ≈ 260K patients from Sutter Palo Alto Medical Foundation Clinics. SUSTain_*M*_ is as accurate as the most accurate baseline (based on [6, 15, 49]), but up to 425× faster (*R* = 5: ≈ 3 seconds by SUSTain_*M*_ vs. ≈ 22 minutes by AILS). Even for a larger target rank (e.g., *R* = 20), SUSTain_*M*_ is 110× faster (≈ 4 minutes by SUSTain_*M*_ vs. ≈ 7 hours by AILS). As compared to a carefully-designed heuristic that performs a scale-and-rounding of the real-valued solution, SUSTain_*M*_ achieves up to 16% higher fit. In summary, SUSTain_*M*_ dominates all other baselines in both time and fit.

SUSTain yields faster and more scalable approaches than baselines achieving comparable fit, as evaluated on both synthetic (publicly-available) and real healthcare datasets. For example, as shown in Figure 1, SUSTain_*M*_ achieves the same level of accuracy as the most accurate baseline **up to 425× faster**. SUSTain_*T*_ can handle large-scale tensor inputs for which the most accurate baseline fails and scales linearly with the number of patients.

SUSTain’s interpretation protocol is particularly meaningful for unsupervised phenotyping: it is easily understood by medical experts who are used to simple and concise, scoring-based descriptions of a patient’s clinical status (e.g., risk scores^1^). While recent work derives risk scores for predictive modeling (supervised learning) [48], our application of SUSTain extracts scores for unsupervised phenotyping based on unlabeled EHR data. In Table 1, we provide a representative phenotype extracted through our method, as part of a case study we performed on phenotyping heart failure patients. The meaningfulness of the phenotype candidates extracted through this case study was confirmed by a cardiologist, who annotated 87% of them as clinically meaningful phenotypes related to heart failure. We summarize our contributions as:

- **Scalable unsupervised scoring:** We propose SUSTain, a fast and scalable approach decomposing integer multi-aspect data into integer scores, preserving the original integer characteristics.
- **SUSTain can handle matrix and tensor input:** We present SUSTain for both matrix (Section 3.1) and tensor (Section 3.2) inputs, through SUSTain_*M*_ and SUSTain_*T*_ methods, respectively.
- **Evaluation on various datasets:** We evaluate both the matrix and tensor versions on both synthetic (publicly-available) and real healthcare datasets.
- **Phenotyping heart failure patients:** The interpretability of the extracted scoring-based phenotypes was confirmed by a cardiologist, who annotated 87% of them as clinically meaningful.

To promote reproducibility, our code is open-sourced and publicly available at: https://github.com/kperros/SUSTain.

## 2 BACKGROUND

In Table 2 we summarize the notations used throughout the paper. Let $x_{0}\in {\mathbb{R}}^{n}$. The *euclidean projection* of ***x***_**0**_ to a set $C\subseteq {\mathbb{R}}^{n}$ is defined as $\Pi_{C}\left(x_{0}\right)=\text{argmin}\left\{{\left\|x-x_{0}\right\|}_{2}^{2}|x\in C\right\}$; thus, it is the problem of determining the vector x* among all ***x*** ∈ *C* which is the closest to ***x***_**0**_ w.r.t. the Euclidean distance [5]. A matrix ***X*** is called *rank-*1 if it can be expressed as the outer product of 2 non-zero vectors: ***X*** = ***x*** ∘ ***y***. The Khatri-Rao Product (KRP) [46] is the “matching column-wise” Kronecker product: for two matrices $U\in {\mathbb{R}}^{M\times R}$, $V\in {\mathbb{R}}^{N\times R}$ their KRP is as follows: $U⊙V=[U(:,1)⊗V(:,1)U(:,2)⊗V(:,2)\ldots U(:,R)⊗V(:,R)]\in {\mathbb{R}}^{MN\times R}$

A *tensor* is a multi-dimensional array. The tensor’s *order* denotes the number of its dimensions, also known as ways or modes (e.g., matrices are 2-order tensors). A *d*-order tensor X is called *rank-*1 if it can be expressed as the outer product of *d* non-zero vectors: ***X*** = ***a***_1_ ∘ ***a***_2_ ∘ ⋯ ∘ ***a***_*d*_. A *fiber* is a vector extracted from a tensor by fixing all modes but one. For example, a matrix column is a mode-1 fiber. A *slice* is a matrix extracted from a tensor by fixing all modes but two. *Matricization*, also called *reshaping* or *unfolding*, logically reorganizes tensors into other forms without changing the values themselves. The mode-*n* matricization of a *d*-order tensor $X\in {\mathbb{R}}^{I_{1}\times I_{2}\times \cdots \times I_{d}}$ is denoted by $X_{\left(n\right)}\in {\mathbb{R}}^{I_{n}\times I_{1}I_{2}\ldots I_{n-1}I_{n+1}\ldots I_{d}}$ and arranges the mode-*n* fibers of the tensor as columns of the resulting matrix. The Matricized-Tensor Times Khatri-Rao Product (MTTKRP) [3] w.r.t. mode-*n* is the matrix multiplication $X_{\left(n\right)}A_{⊙}^{\left(-n\right)}$, where $A_{⊙}^{\left(-n\right)}$ corresponds to the Khatri-Rao product of all the modes except the *n*-th. MTTKRP is the bottleneck operation in many sparse tensor algorithms.

## 3 THE SUSTAIN FRAMEWORK

First we present SUSTain for matrix input. Then, we describe how SUSTain can be extended for general high-order tensor input. Finally, we provide our interpretation protocol of SUSTain for unsupervised phenotyping.

### 3.1 SUSTain for matrix input

#### Model:

For an integer input matrix $X\in {\mathbb{Z}}_{+}^{M\times N}$ and a certain target rank *R*, the problem can be defined as:

(2) $$\text{min}\left\{{\left\|X-U\;\Lambda \;V^{T}\right\|}_{F}^{2}|U\in {\mathbb{Z}}_{\tau }^{M\times R},V\in {\mathbb{Z}}_{\tau }^{N\times R},\Lambda \in {\mathbb{Z}}_{+}^{R\times R}\right\}$$

where ${\mathbb{Z}}_{\tau }=\left\{0,1,\ldots ,\tau \right\}$ is the set of nonnegative integers up to *τ*, ${\mathbb{Z}}_{+}=\left\{1,2,\ldots ,\infty \right\}$ is the set of positive integers and **Λ** is a diagonal *R*-by-*R* matrix. The above problem can be also formulated as ${\left\|X-\sum_{r=1}^{R}\lambda (r)\;U(:,r)\;V{\left(:,r\right)}^{T}\right\|}_{F}^{2}$ where ***λ***(*r*) = **Λ**(*r*, *r*). The reason for having ***λ***(*r*) is to absorb any scaling of each *r*-th rank-1 component, since the entries of ***U*** and ***V*** factors are upper bounded by *τ*. Note that the ***λ***(*r*) values cannot be simply obtained through normalization as in the corresponding real-valued models (e.g., NMF [25, 35]), due to the integer constraints. Finally, note that the integer set ${\mathbb{Z}}_{\tau }$ can easily vary for different factor matrices and even allow negative integers; this can also happen for the input matrix ***X***. The formulation in Problem (2) favors simplicity of presentation and matches the need of phenotyping applications.

#### Fitting Algorithm:

We employ an alternating updating scheme to tackle the non-convex optimization Problem (2). Our scheme leads to optimal solutions to each one of the sub-problems in an efficient manner, while organizing the order of updates so as to promote re-use of already computed intermediate results.

We follow the intuition behind the Hierarchical Alternating Least Squares (HALS) framework, which enables isolating and solving for each *k*-th rank-1 component separately. Thus, Problem (2) gives:

(3) $$\text{min}\left\{{\left\|\underset{R_{k}}{\underset{︸}{X-\sum_{r=1,r\neq k}^{R}\lambda (r)\;U(:,r)\;V{\left(:,r\right)}^{T}}}-\lambda (k)\;U(:,k)\;V{\left(:,k\right)}^{T}\right\|}_{F}^{2}|U\in {\mathbb{Z}}_{\tau }^{M\times R},V\in {\mathbb{Z}}_{\tau }^{N\times R},\Lambda \in {\mathbb{Z}}_{+}^{R\times R}\right\}$$

where ***R***_*k*_ corresponds to the “residual matrix” and is considered fixed when solving for the *k*-th rank-1 component. The objective can be written as [30]:

$$J={\left\|R_{k}\right\|}_{F}^{2}+\lambda^{2}(k){\left\|U(:,k)\right\|}_{2}^{2}{\left\|V(:,k)\right\|}_{2}^{2}-2\;\lambda (k)U{\left(:,k\right)}^{T}R_{k}\;V(:,k)$$

We set:

$$\partial J/\partial \lambda (k)=2\;\lambda (k){\left\|U(:,k)\right\|}_{2}^{2}{\left\|V(:,k)\right\|}_{2}^{2}-2\;U{\left(:,k\right)}^{T}R_{k}\;V(:,k)=0$$

and obtain:

$$\lambda_{k}^{*}≔\frac{U{\left(:,k\right)}^{T}\;R_{k}\;V(:,k)}{{\left\|U(:,k)\right\|}_{2}^{2}\;{\left\|V(:,k)\right\|}_{2}^{2}}$$

If ***U*** (:, *k*)^*T*^ ***R***_*k*_ ***V*** (:, *k*) > 0 then the minimum value of *J* for $\lambda (k)\in {\mathbb{Z}}_{+}$ is obtained at $max\left(1,\text{round}\left(\lambda_{k}^{*}\right)\right)$ where *round*() rounds to the nearest integer. If ***U*** (:, *k*)^*T*^ ***R***_*k*_ ***V*** (:, *k*) ≤ 0, then the minimum objective value for $\lambda (k)\in {\mathbb{Z}}_{+}$ is attained at ***λ***(*k*) = 1. Combining these two cases, the optimal $\lambda (k)\in {\mathbb{Z}}_{+}$ is given by:

(4) $$\lambda (k)\leftarrow max\left(1,\text{round}\left(\frac{U{\left(:,k\right)}^{T}\;R_{k}\;V(:,k)}{{\left\|U(:,k)\right\|}_{2}^{2}\;{\left\|V(:,k)\right\|}_{2}^{2}}\right)\right)$$

In practice, ***R***_*k*_ may be large (*M* × *N*) and dense, even if the input is sparse (as happens in our main motivating application); thus its explicit materialization should be avoided [16, 27]. Expanding the above expression gives:

(5) $$\lambda (k)\leftarrow max\left(1,\text{round}\left(\lambda (k)+\frac{V{\left(:,k\right)}^{T}\;\left({\left[X^{T}U\right]}_{:,k}-V\;\Lambda {\left[U^{T}\;U\right]}_{:,k}\right)}{{\left[U^{T}\;U\right]}_{k,k}{\left[V^{T}\;V\right]}_{k,k}}\right)\right)$$

Next, solving Problem (3) for ***V*** (:, *k*) gives:

(6) $$\text{min}\left\{{\left\|R_{k}-\lambda (k)\;U(:,k)\;V{\left(:,k\right)}^{T}\right\|}_{2}^{2}|V(:,k)\in {\mathbb{Z}}_{\tau }^{N}\right\}$$

To solve the above, we apply the Optimal Scaling Lemma [8] for the integer constraint. This Lemma states that for any set *C* of constraints imposed on ***b***, it holds that:

$$\text{min}\left\{{\left\|Y-x\;b^{T}\right\|}_{2}^{2}|b\in C\right\}=\Pi_{C}(\beta )$$

where $\beta =\frac{x^{T}\;Y}{x^{T}\;x}$ is the unconstrained solution to the above problem. This means that the optimal solution of the constrained problem is simply the projection of the unconstrained solution onto the constraint set *C*. Thus, the optimal solution of Problem (6) is:

(7) $$V(:,k)\leftarrow \Pi_{{\mathbb{Z}}_{\tau }^{N}}\left(\frac{R_{k}^{T}\;U(:,k)}{{\left[U^{T}\;U\right]}_{k,k}\;\lambda (k)}\right)$$

Since ${\mathbb{Z}}_{\tau }^{N}$ is the Cartesian product of subsets of the real line, i.e., ${\mathbb{Z}}_{\tau }^{N}=\underset{N\text{ times}}{\underset{︸}{{\mathbb{Z}}_{\tau }\times {\mathbb{Z}}_{\tau }\times \cdots \times {\mathbb{Z}}_{\tau }}}$ we can take

(8) $$\Pi_{{\mathbb{Z}}_{\tau }^{N}}(V(:,k))=\left[\Pi_{{\mathbb{Z}}_{\tau }}(V(1,k)),\ldots ,\Pi_{{\mathbb{Z}}_{\tau }}(V(N,k))\right]$$

thus project each scalar coordinate individually. For a real-valued scalar *α*, projecting onto ${\mathbb{Z}}_{\tau }$ gives [47]:

(9) $$\Pi_{{\mathbb{Z}}_{\tau }}(\alpha )=min(max(\text{round}(\alpha ),0),\tau )$$

Finally, expanding ***R***_*k*_ in Expression (7), combining with (8), (9) and setting:

(10) $$b\leftarrow V(:,k)+\frac{{\left[X^{T}\;U\right]}_{:,k}-V\;\Lambda \;{\left[U^{T}\;U\right]}_{:,k}}{{\left[U^{T}\;U\right]}_{k,k}\;\lambda (k)}$$

gives the optimal solution for Problem (6):

(11) V(:,k)←min(max(round(b),0),τ)

where *min*(), *max*(), *round*() are taken element-wise.

Having derived the updates for ***λ***(*k*), ***V*** (:, *k*), in Relations (5) and (11) respectively, we remark that the computationally expensive intermediate results [***X***^*T*^ ***U***] and [***U***^*T*^ ***U***] are shared between them. To exploit that, we choose to successively update ***λ***(*k*) and ***V***(:, *k*) during the same iteration and iterate ∀*k* ∈ {1, … , *R*}. As a result of the proposed update order, the only non-negligible additional operation in order to compute both ***λ***(*k*) and***V*** (:, *k*) is to re-compute ***t*** ≔ ***V* Λ *U***^*T*^ ***U***]_:, *k*_ after having updated ***λ***(*k*).

Re-computing ***t*** can be further optimized by observing that only the contribution of the *k*-th component ***t***_***k***_ ≔ ***V*** (:, *k*) * ***λ***(*k*) [***U***^*T*^ ***U***]_*k*, *k*_ has to be adjusted. Thus, we can store ***t*** and ***t_k_***, compute ***λ***′(*k*), and then adjust ***t*** as: ***t*** ← ***t*** − ***t***_***k***_ + (***V*** (:, *k*) * ***λ***′(*k*) [***U***^*T*^ ***U***]_*k*, *k*_).

Updating ***U*** (:, *k*) can be executed in symmetric fashion to ***V*** (:, *k*). In Algorithm 1, we present our main procedure to update both the factor matrices ***U*** and ***V*** and ***λ*** values in an alternating fashion. In Algorithm 2, we provide the definition of SUSTain_Update_Factor which updates a single factor (denoted as ***F***) and the vector ***λ***.

**Algorithm 1:** SUSTain_*M*_

**Require:** $X\in {\mathbb{R}}^{M\times N}$, target rank *R* and upper bound *τ*
**Ensure:** $U\in {\mathbb{Z}}_{\tau }^{M\times R}$, $V\in {\mathbb{Z}}_{\tau }^{N\times R}$, $\lambda \in {\mathbb{Z}}_{+}^{R}$
1: Initialize ***U***, ***V***, **Λ**
2: **while** convergence criterion is not met **do**
3: ***F*** ← ***U***, ***M*** ← ***X V***, ***C*** ← ***V***^*T*^ ***V***
4: [***U***, ***λ***] = SUSTain_Update_Factor(***F***, ***M***, ***C***, ***λ***, *R*, *τ*)
5: ***F*** ← ***V***, ***M*** ← ***X***^*T*^ ***U***, ***C*** ← ***U***^*T*^ ***U***
6: [***V***, ***λ***] = SUSTain_Update_Factor(***F***, ***M***, ***C***, ***λ***, *R*, *τ*)
7: **end while**

**Algorithm 2:** SUSTain_Update_Factor(***F***, ***M***, ***C***, ***λ***, *R*, *τ*)

**Require:** $F\in {\mathbb{Z}}_{\tau }^{I\times R}$, $M\in {\mathbb{R}}^{I\times R}$, $C\in {\mathbb{R}}^{R\times R}$, $\lambda \in {\mathbb{Z}}_{+}^{R}$, target rank *R* and upper bound *τ*
**Ensure:** $F\in {{\mathbb{Z}}_{\tau }}^{I\times R}$, $\lambda \in {\mathbb{Z}}_{+}^{R}$
1: **for** *k* = 1, … , *R* **do**
2: ***t*** ← ***F*** (***λ*** * ***C***(:, *k*))
3: ***t***_***k***_ ← ***F*** (:, *k*) * ***λ***(*k*) ***C***(*k*, *k*)
4: $\alpha \leftarrow \lambda (k)+\frac{F{\left(:,k\right)}^{T}(M(:,k)-t)}{C(k,k){\left[F^{T}\;F\right]}_{k,k}}$
5: *λ*(*k*) ← *max* (1, *round* (*α*))
6: ***t*** ← ***t*** − ***t***_***k***_ + (***F*** (:, *k*) * ***λ***(*k*) ***C***(*k*, *k*))
7: $b\leftarrow F(:,k)+\frac{M(:,k)-t}{C(k,k)\lambda (k)}$
8: ***F*** (:, *k*) ← *min* (*max* (*round* (***b***), 0), *τ*)
9: **end for**

#### Computational Complexity:

The asymptotic cost of executing Algorithm 2 is 2*R*^2^*I* flops (i.e., floating-point operations), ∀*R* > 5. This step costs 2*R*^2^*N* when updating ***V*** and 2*R*^2^*M* when updating ***U***. In Algorithm 1, assuming the input ***X*** is sparse, the cost of each one of ***X V*** and ***X***^*T*^ ***U*** is 2 *nnz* (***X***) *R* flops. Also, computing ***V***^*T*^ ***V*** and ***U***^*T*^ ***U*** cost 2*R*^2^*N* and 2*R*^2^*M* flops respectively. Thus, the total cost is: 4*R* (*nnz*(***X***) + (*M* + *N*)*R*) flops.

### 3.2 SUSTain for tensor input

#### Model:

For a tensor $X\in {\mathbb{R}}^{I_{1}\times I_{2}\times \ldots I_{d}}$ of order *d* and a certain target rank *R*, the problem can be defined as

(12) $$\text{min}\left\{{\left\|X-\overset{R}{\underset{r=1}{\sum }}\lambda (r)\;A^{\left(1\right)}(:,r)\circ \ldots \circ A^{\left(d\right)}(:,r)\right\|}_{F}^{2}|A^{\left(n\right)}\in {\mathbb{Z}}_{\tau }^{I_{n}\times R},\lambda (r)\in {\mathbb{Z}}_{+}\right\}$$

where *n* = {1, … , *d*}, ${\mathbb{Z}}_{\tau }=\left\{0,1,\ldots ,\tau \right\}$ is the set of nonnegative integers up to *τ* and ${\mathbb{Z}}_{+}=\left\{1,2,\ldots ,\infty \right\}$ is the set of positive integers. Our model is an extension of SUSTain_*M*_ presented in Section 3.1 for high-order tensors. It can be viewed as a constrained version of the CP tensor model [9, 18, 20].

#### Fitting Algorithm:

Similarly to the matrix case, we set:

$$R_{k}≔X-\overset{R}{\underset{r=1,r\neq k}{\sum }}\lambda (r)\;A^{\left(1\right)}(:,r)\circ \ldots \circ A^{\left(d\right)}(:,r)$$

Thus, Problem (12) becomes:

(13) $$\text{min}\left\{{\left\|R_{k}-\lambda (k)\;A^{\left(1\right)}(:,k)\circ \ldots \circ A^{\left(d\right)}(:,k)\right\|}_{F}^{2}|A^{\left(n\right)}\in {\mathbb{Z}}_{\tau }^{I_{n}\times R},\lambda (k)\in {\mathbb{Z}}_{+}\right\}$$

We matricize the above expression w.r.t. mode-*n* and utilize the fact that the mode-*n* matricization of a rank-1 tensor ***b***_1_ ○ ⋯ ○ ***b***_*d*_ can be expressed as ***b***_*n*_ (***b***_d_ ⊗ ⋯ ⊗ ***b***_*n*+1_ ⊗ ***b***_*n*−1_ ⊗ ⋯ ⊗ *b*_1_)^*T*^ [17]:

(14) $$\text{min}\left\{{\left\|R_{k(n)}-\lambda (k)A^{\left(n\right)}(:,k){\left(A^{\left(d\right)}(:,k)⊗\cdots ⊗A^{\left(n+1\right)}(:,k)⊗A^{\left(n-1\right)}(:,k)⊗\cdots ⊗A^{\left(1\right)}(:,k)\right)}^{T}\right\|}_{F}^{2}|A^{\left(n\right)}\in {\mathbb{Z}}_{\tau }^{I_{n}\times R},\lambda (k)\in {\mathbb{Z}}_{+}\right\}$$

We set $A_{⊙}^{\left(-n\right)}≔A^{\left(d\right)}⊙\cdots ⊙A^{\left(n+1\right)}⊙A^{\left(n-1\right)}⊙\cdots ⊙A^{\left(1\right)}$ as the Khatri-Rao Product of all the factor matrices except the *n*-th and

(15) $$C^{\left(-n\right)}≔A^{{\left(d\right)}^{T}}A^{\left(d\right)}*\cdots *A^{{\left(n+1\right)}^{T}}A^{\left(n+1\right)}*A^{{\left(n-1\right)}^{T}}A^{\left(n-1\right)}*\cdots *A^{{\left(1\right)}^{T}}A^{\left(1\right)}$$

as the Hadamard product of the Gram matrices of all the factor matrices except the *n*-th. Then, Objective (14) becomes

(16) $$\text{min}\left\{{\left\|R_{k(n)}-\lambda (k)A^{\left(n\right)}(:,k)A_{⊙}^{\left(-n\right)}{\left(:,k\right)}^{T}\right\|}_{F}^{2}|A^{\left(n\right)}\in {\mathbb{Z}}_{\tau }^{I_{n}\times R},\lambda (k)\in {\mathbb{Z}}_{+}\right\}$$

Solving the above for ***λ***(*k*) can be handled equivalently to the corresponding matrix case (Relation (4)). Thus, the optimal solution for $\lambda (k)\in {\mathbb{Z}}_{+}$ is:

(17) $$\lambda (k)\leftarrow max\left(1,\;\text{round }\left(\frac{A^{\left(n\right)}{\left(:,k\right)}^{T}R_{k(n)}A_{⊙}^{\left(-n\right)}(:,k)}{{\left\|A^{\left(n\right)}(:,k)\right\|}_{2}^{2}\;{\left\|A_{⊙}^{\left(-n\right)}(:,k)\right\|}_{2}^{2}}\right)\right)$$

By exploiting that [29]:

$${\left\|A_{⊙}^{\left(-n\right)}(:,k)\right\|}_{2}^{2}=A_{⊙}^{\left(-n\right)}{\left(:,k\right)}^{T}A_{⊙}^{\left(-n\right)}(:,k)={\left[A_{⊙}^{{\left(-n\right)}^{T}}A_{⊙}^{\left(-n\right)}\right]}_{k,k}=C^{\left(-n\right)}(k,k)$$

and expanding:

(18) $$R_{k(n)}A_{⊙}^{\left(-n\right)}(:,k)=M^{\left(n\right)}(:,k)-A^{\left(n\right)}\;\Lambda \;C^{\left(-n\right)}(:,k)+\lambda (k)C^{\left(-n\right)}(k,k)A^{\left(n\right)}(:,k)$$

where ***M***^(*n*)^(:, *k*) is the Matricized-Tensor Times Khatri-Rao Product (MTTKRP) [3] operation w.r.t. mode *n*, we get the optimal solution for $\lambda (k)\in {\mathbb{Z}}_{+}$ as:

(19) $$\lambda (k)\leftarrow max\left(1,\;\text{round }\left(\lambda (k)+\frac{A^{\left(n\right)}{\left(:,k\right)}^{T}\left(M^{\left(n\right)}(:,k)-A^{\left(n\right)}\;\Lambda \;C^{\left(-n\right)}(:,k)\right)}{C^{\left(-n\right)}(k,k){\left[A^{{\left(n\right)}^{T}}\;A^{\left(n\right)}\right]}_{k,k}}\right)\right)$$

Next, we transpose the Objective (16) and solving for ***A***^(*n*)^(:, *k*) can be handled as in the matrix case (Relation (7)) through the(Optimal Scaling Lemma [8]. Thus, the optimal $A^{\left(n\right)}(:,k)\in {\mathbb{Z}}_{\tau }^{I_{n}}$ is given by:

(20) $$A^{\left(n\right)}(:,k)\leftarrow \Pi_{{\mathbb{Z}}_{\tau }^{In}}\left(\frac{R_{k(n)}A_{⊙}^{\left(-n\right)}(:,k)}{C^{\left(-n\right)}(k,k)\;\lambda (k)}\right)$$

Finally, combining Equation (18) into the above gives:

(21) $$A^{\left(n\right)}(:,k)\leftarrow \Pi_{{\mathbb{Z}}_{\tau }^{In}}\left(A^{\left(n\right)}(:,k)+\frac{M^{\left(n\right)}(:,k)-A^{\left(n\right)}\;\Lambda \;C^{\left(-n\right)}(:,k)}{C^{\left(-n\right)}(k,k)\;\lambda (k)}\right)$$

Note the direct correspondence of the above formulations for ***λ***(*k*), ***A***^(*n*)^(:, *k*) with the core update Algorithm 2 we used for the matrix case. If we set ***F*** ← ***A***^(*n*)^, ***M*** ← ***M***^(*n*)^, ***C*** ← ***C***^(−*n*)^ then we can simply use Algorithm 2 to update a single factor ***A***^(*n*)^ and the ***λ*** values. Also, we can exploit the development of existing scalable software libraries computing the bottleneck MTTKRP kernel for sparse data efficiently [3]. In Algorithm 3, we summarize the operations of our methodology for tensor input.

#### Computational Complexity:

Updating the *n*-th mode in Algorithm 3 requires: 3 R nnz(X) flops to compute the MTTKRP using state-of-the-art libraries for sparse tensors [3], 2 *R*^2^ *I*_*n*_ flops to compute $A^{{\left(n\right)}^{T}}\;A^{n}$ and (*d* − 1) *R*^2^ flops to update ***C***^(−*n*)^ as in Equation (15). As discussed in the matrix case, the dominant cost of Algorithm 2 is 2 *R*^2^ *I*_*n*_ flops. Overall, Algorithm 3 requires: $3\;d\;R\;nnz(X)+4\;R^{2}\sum_{n=1}^{d}I_{n}+d(d-1)R^{2}$ flops. In our experiments, the first term, thus the computation of MTTKRP, dominates the total cost.

**Algorithm 3:** SUSTain_*T*_

**Require:** $X\in {\mathbb{R}}^{I_{1}\times I_{2}\times \ldots I_{d}}$, target rank *R* and upper bound *τ*
**Ensure:** $A^{\left(n\right)}\in {\mathbb{Z}}_{\tau }^{I_{n}\times R}$, with *n* ∈ {1, … , *d*}, $\lambda \in {\mathbb{Z}}_{+}^{R}$
1: Initialize ***A***^(***n***)^, ***λ***
2: **while** convergence criterion is not met **do**
3: **for** *n* = 1, … , *d* **do**
4: $M^{\left(n\right)}\leftarrow X_{\left(n\right)}A_{⊙}^{\left(-n\right)}$ // *MTTKRP*
5: Compute ***C***^(−*n*)^ as in Relation (15)
6: [***A***^(*n*)^, ***λ***] = SUSTain_Update_Factor(***A***^(*n*)^, ***M***^(*n*)^, ***C***^(−*n*)^, ***λ***, *R*, *τ*)
7: **end for**
8: **end while**

### 3.3 Interpretation for phenotyping

Given the EHRs of a certain cohort, we form a patient-by-diagnoses matrix ***X***, whose ***X***(*i*, *j*) cell is the number of encounters of patient *i* where encounter diagnosis *j* was recorded. In that case, the patient membership vector ***U*** (*i*, :) of SUSTain_*M*_ provides the distinct levels of frequency of each one of the *R* phenotypes throughout the medical history of the *i*-th patient. Likewise, each column ***V*** (:, *r*) indicates the frequency levels of each medical feature w.r.t. the *r*-th phenotype. Table 1 summarizes a phenotype example that accounts for the largest share of heart failure patients. Finally, due to the integer box (i.e., {0, … , *τ*}) constraints employed on the factor matrices, we can interpret the integer ***λ***(*r*) values as scaling up the input encounter counts for the *r*-th phenotype. Thus, phenotypes with higher ***λ***(*r*) values are expected to describe more persistent medical conditions, with higher number of associated encounters.

The above interpretation can be extended to the tensor case. Consider a tensor X whose X(i,j,k) cell defines the count of encounters of patient *i* where medication *k* was ordered for the patient with diagnosis *j* as the order indication. Factorizing this tensor using SUSTain_*T*_ yields a patient factor ***A***^(1)^ which can be interpreted similarly to the ***U*** factor in the matrix case. Also, the factor matrices ***A***^(2)^, ***A***^(3)^ corresponding to diagnosis and medication or procedure phenotypes can be interpreted similarly to the ***V*** factor in the matrix case. The same applies to the ***λ***(*r*) model values.

## 4 EXPERIMENTS

### 4.1 Setup

#### 4.1.1 Description of datasets.

Table 3 summarizes statistics for the datasets used.

##### Sutter:

This dataset corresponds to EHRs from Sutter Palo Alto Medical Foundation (PAMF) Clinics. The patients are 50 to 80 years old adults chosen for a heart failure study [11]. To form a patient-by-diagnosis matrix input, we extracted the number of encounter records with a specific diagnosis for each patient. To form a patient-diagnosis-medication tensor input, we used the medication orders, reflecting the ordered medications and the indicated diagnosis. We adopt standard medical concept groupers to group the available ICD-9 diagnosis codes [45] into Clinical Classification Software (CCS) [1] diagnostic categories (level 4). We also group the normalized drug names (i.e., combining all branded names and the generic name for a medication) based on unique therapeutic subclasses using the Anatomical Therapeutic Chemical Classification System [39].

##### CMS:

We used a *publicly-available* CMS Linkable 2008–2010 Medicare Data Entrepreneurs’ Synthetic Public Use File (DE-SynPUF)^2^ that contains three years of claim records synthesized (i.e., to protect privacy) from 5% of the 2008 Medicare population. CMS creates twenty 5% subsamples of the claims data. We used the carrier claims data available from DE-SynPUF for the patients belonging to Samples 1 & 2. We increase the number of samples (i.e., number of patients) considered for the experiments related to assessing scalability. We used the diagnostic code information to build the input matrix and the diagnoses and procedures recorded to build the input tensor. In particular, we group the available ICD-9 diagnosis codes [45] into CCS [1] diagnostic categories (level 4) and use the CCS flat code grouper [1] to transform the CPT procedure codes available into procedure categories.

#### 4.1.2 Baselines.

Below, we describe our efforts to design competitive baseline methods producing the target models in Problems 2 and 12, for the matrix and the tensor cases respectively.

##### Round:

This baseline rounds the factor matrices from nonnegative matrix/tensor factorization. In the matrix case, we used the implementation of Nonnegative Matrix Factorization (NMF) [27, 28] and projected all the entries of the resulting factor matrices to ${\mathbb{Z}}_{\tau }$. We also set ***λ*** to an all-ones vector as NMF typically does not have the diagonal matrix **Λ**. A typical issue of naively rounding NMF solutions is that values that are lower than 0.5 are rounded to 0, so a potentially large part of model information can be lost.

In the tensor case, we used the CP-ALS algorithm as in the Tensor Toolbox [4], adjusted to impose non-negativity constraints [28] on the factor matrices. Also, in contrast to the NMF case, CP-ALS produces a ***λ*** vector of nonnegative real values. In order to alleviate the effect of zeroing out values less than 0.5 we compute the cube root of the ***λ*** vector element-wise and form a vector $\hat{\lambda }$. Then, we absorb this scaling in the factor matrices by multiplying $A^{\left(n\right)}\text{diag}(\hat{\lambda }),\forall n=\left\{1,\ldots ,d\right\}$ where *d* is the input tensor’s order. Finally, we set ***λ*** to an all-ones vector and project all the entries of the resulting factor matrices to ${\mathbb{Z}}_{\tau }$.

##### Scale-and-round:

We design a more sophisticated scale-and-rounding heuristic which scales the factor matrices of the real-valued solutions before performing the rounding. This step further alleviates the problem of zeroing out values less than 0.5.

In the matrix case, we define the scaling factor *γ*_2_(*j*) = *τ*/*max*(***V*** (:, *j*)). Then, $\tilde{V}(:,j)=\text{round}\left(\gamma_{2}(j)V(:,j)\right)$. Similarly, we define *γ*_1_(*j*) = *τ*/*max*(***U*** (:, *j*)). Then, $\tilde{U}(:,j)=\text{round}\left(\gamma_{1}(j)U(:,j)\right)$. Those steps scale-up the maximum value of factor matrix column to reach the upper bound *τ*. Then, this excess scaling is absorbed into ***λ*** as: ***λ*** = *round*(1/*γ*_1_*γ*_2_).

In the tensor case, we absorb the scaling of the ***λ*** output of the real-valued solution into the factor matrices as in “Round”, and extend the Scale-and-round matrix approach accordingly.

##### AILS: Alternating Integer Least Squares approach:

We used the Integer Least Squares (ILS) with box constraints approach which is proposed in [6, 49]. This approach was recently unified within an Integer Matrix Factorization framework [15]. We exploit the redundancy among ILS problems targeting the same factor matrix, so that the QR factorization in the reduction phase is only computed once. Note that solving general ILS problems is NP-hard [15], which is reflected in the runtime of this method in the experiments. We enabled the extraction of the integer ***λ*** values through an ILS by noticing that vectorizing the original problem as

$$\text{min}\left\{{\left\|vec\left(U\;\Lambda \;V^{T}\right)-vec(X)\right\|}_{F}^{2}|\Lambda \in {\mathbb{Z}}_{+}^{R\times R}\right\}$$

can be transformed to [7]: $\text{min}\left\{{\left\|\left(V⊙U)\lambda -vec(X\right)\right\|}_{F}^{2}|\lambda \in {\mathbb{Z}}_{+}^{R}\right\}$ which gives the ILS to solve for. Note that we attempted to extend this approach for tensor input; however, the materialization of the Khatri-Rao product of all the factor matrices failed due to out of memory problems even for the smallest target rank for both of the datasets used. To illustrate the magnitude of this issue, the size needed for the Khatri-Rao product of all factor matrices for Sutter data and *R* = 5 is: 248347 * 552 * 555 * 5 * 8 bytes ≈ 3 Terabytes.

#### 4.1.3 Evaluation metrics.

We evaluate the methods under comparison in terms of the trade-off between execution time and accuracy for various target ranks considered (*R* = {5, 10, 20, 40}). Accuracy is measured in terms of fit: $1-{\left\|X-\hat{X}\right\|}_{F}/{\left\|X\right\|}_{F}$, where $\hat{X}$ the re-constructed through the model factors (this extends trivially to the tensor case); fit can be considered as the the proportion of data explained by the model.

#### 4.1.4 Initialization details.

In all experiments, when we compare SUSTain and AILS, we provide them with the same initialization.

Regarding the accuracy-time trade-off evaluation, we initialize with several schemes and for each method we choose the one providing the highest fit. The schemes are the following: a) round heuristic, b) scale-and-round heuristic, c) random: random initialization with integers within the required range and ***λ*** set to all-ones vector, d) random & sampling: random initialization of the patients factor and sampling from the input data to populate the rest of the factors. In the matrix case, we initialize each *j*-th column of ***V*** by random sampling of input patient vectors and scaling them to lie on ${\mathbb{Z}}_{\tau }$ if needed. In the tensor case, for each sampled slice X(i,:,:), we populate each *j*-th component of ***A***^(2)^, ***A***^(3)^ by sampling the row and column of X(i,:,:) with the maximum sum. Note that when we measure execution time for each approach, we do take into account the time spent for its initialization.

In the scalability evaluation, we initialize each method with the random & sampling scheme (d) described above; this provided better starting points than using pure random initialization. For this experiment, we ignore the initialization time, since we want to focus on the methods’ scalability behavior.

#### 4.1.5 Implementation details.

We used MatlabR2017b for our implementations, along with functionalities for sparse tensors from the “Tensor Toolbox” [4] and for nonnegative matrix factorization from the “nonnegfac-matlab” [27] toolbox. The ILS solver we use for the AILS baseline is included in the state-of-the-art MILES software [10].

The zero-lock problem refers to the case when a single column is zeroed out, thus zeroing out an entire rank-1 component of the solution. To avoid that in our scheme, we add the smallest perturbation possible (+1) to a randomly-chosen coordinate of the vector zeroed out.

In both SUSTain and AILS, we break the iterations when the successive difference of the objective drops below 1*e* − 4. Finally, the parameter *τ* is set to 5 driven by discussions with medical experts and similarity to many medical scoring systems.

#### 4.1.6 Hardware.

We conducted our experiments on a server running Ubuntu 14.04 with 1TB of RAM and four Intel E5–4620 v4 CPU’s with a maximum clock frequency of 2.10GHz. Each of the processors contains 10 cores with 2 threads each.

### 4.2 Matrix case experiments

#### Accuracy-Time trade-off:

In Figure 1, we showcase the accuracy-time trade-off regarding the Sutter PAMF dataset. SUSTain_*M*_ is at least 60× faster (*R* = 40) than the most accurate baseline (AILS). For *R* = 5, SUSTain_*M*_ achieves 425× speedup over AILS: as compared to the ≈ 22 **minutes** spent by AILS, our approach executes in ≈ **3 seconds** for the same level of accuracy. Even for *R* = {10, 20} SUSTain_*M*_ achieves 98× and 110× faster computations than AILS. At the same time, SUSTain_*M*_ achieves up to 16% higher fit than the scale-and-round heuristic, operating on comparable running times. Note that for *R* = 5, our approach is even faster (and more accurate) than the scale-and-round baseline as well, since initializing with random factors provided a better final fit than initializing with the scale-and-round result. We also remark that the naive round heuristic achieves a fit of zero, which is a by-product of zeroing out the majority of the model factor elements.

**Figure 2:**
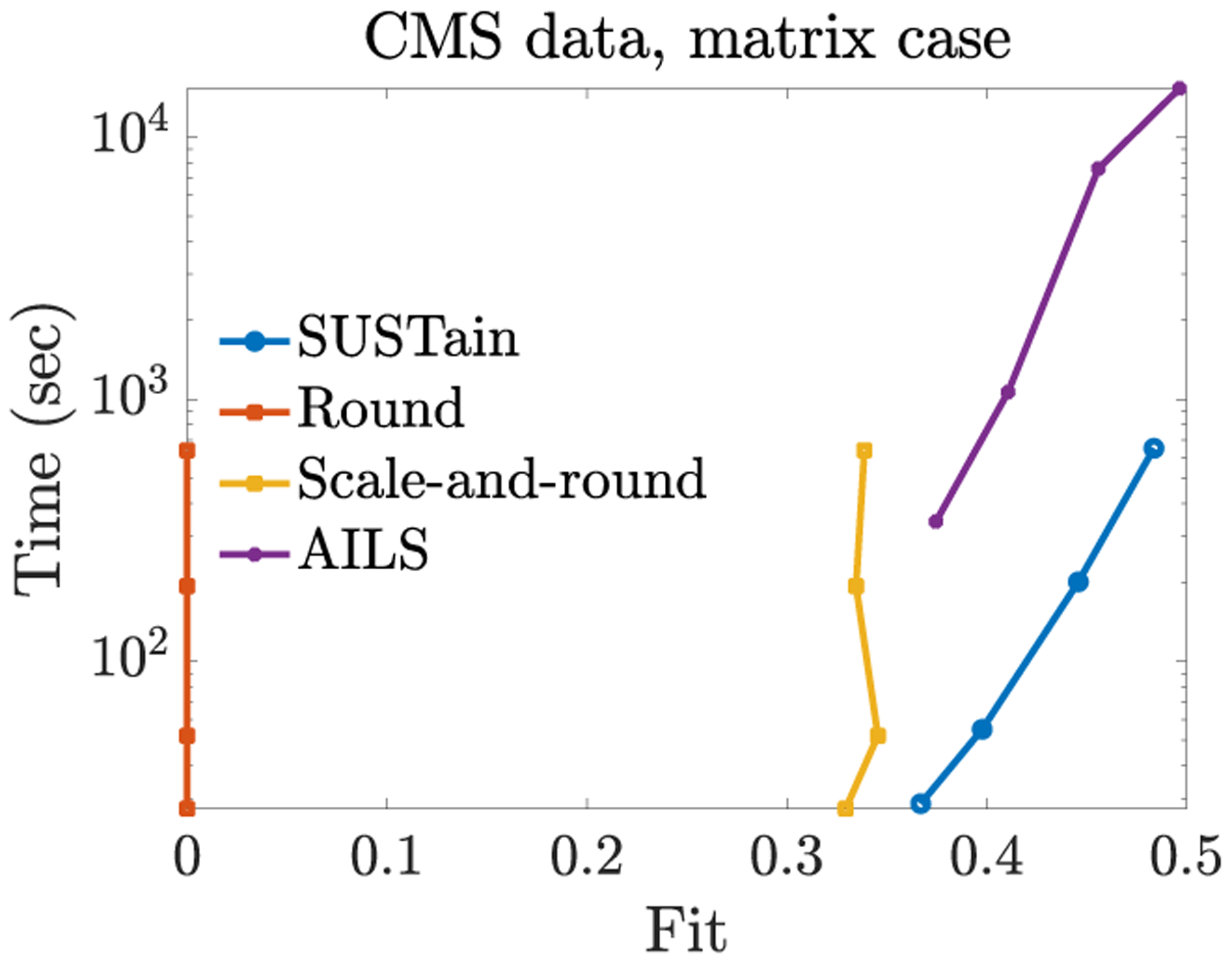
Fit (range [0, 1]) vs time trade-off for varying target number of phenotypes *R* = {5, 10, 20, 40} for the CMS matrix input. SUSTain_*M*_ is at least an order of magnitude faster than the most accurate baseline (up to 38× faster for *R* = 20), while achieving the same level of accuracy. Also, SUSTain_*M*_ achieves up to 14% higher fit over scale-and-rounding heuristics.

In Figure 2, we provide the results of the same experiment regarding the CMS dataset. For the same level of accuracy, SUSTain_*M*_ is at least an order of magnitude faster than AILS, and up to 38× faster for *R* = 20. It also achieves up to 14% higher fit over the scale-and-rounding heuristic for comparable execution time.

#### Scaling for larger number of patients:

In Table 4, for fixed *R* = 10, we measure a single iteration’s time for increasing subsets of CMS patients. The NMF execution time is considered for Round and Scale-and-round heuristics, since their post-processing cost is negligible. SUSTain_*M*_ can execute very fast (a single iteration in ≈ 3 seconds) even for ≈ 985 thousand patients.

### 4.3 Tensor case experiments

#### Accuracy-Time trade-off:

**Figure 3:**
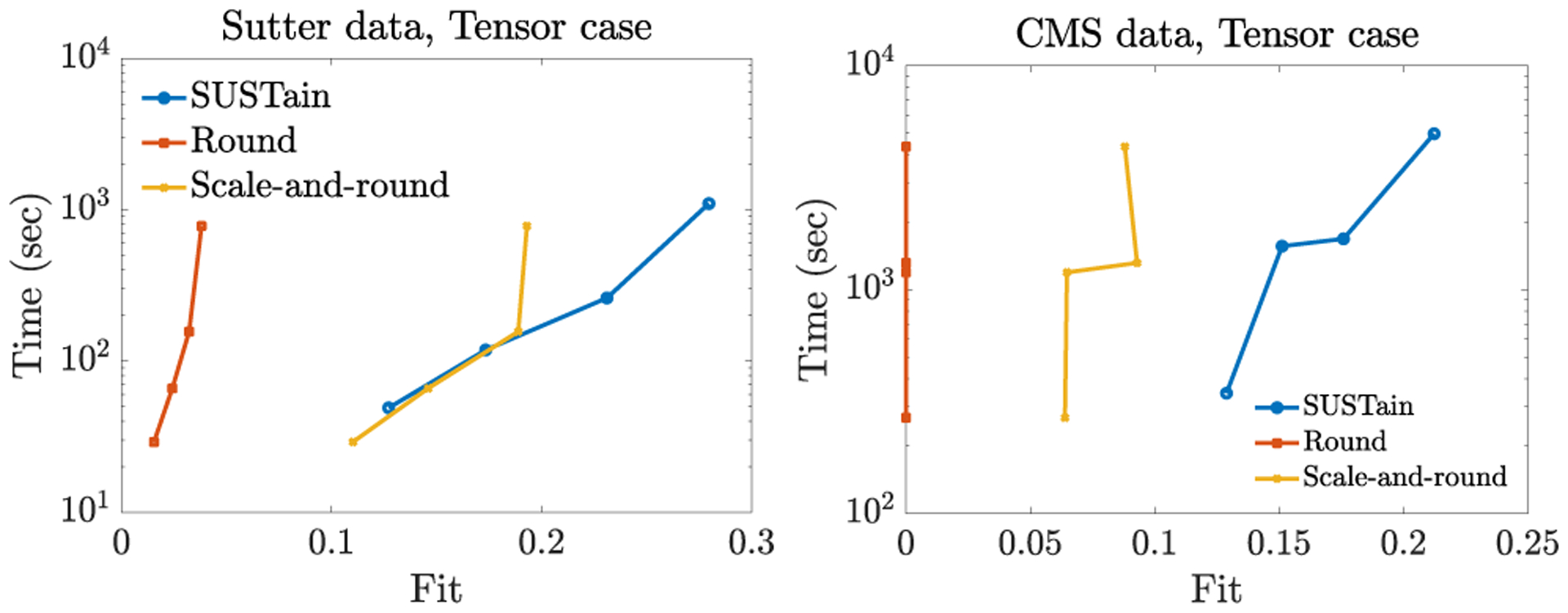
Fit (range [0, 1] vs time trade-off for varying target number phenotypes *R* = {5, 10, 20, 40} for the Sutter and the CMS tensor input. SUSTain_*T*_ achieves up to 9% and 12% higher fit respectively over scale-and-rounding heuristics.

In Figure 3, we provide the fit-time trade-off for varying target rank of our input tensor datasets. As discussed in Section 4.1.2, the extension of AILS approach to tensors cannot scale for any dataset or target rank considered. Overall, SUSTain_*T*_ achieves up to 9% and 12% increase in fit over the scale- and-round heuristic w.r.t. the Sutter PAMF and CMS datasets respectively. Note that the fit of the scale-and-round approach decreases for successively increasing target rank values (e.g., transitioning from *R* = 20 to *R* = 40 for CMS data). This indicates that heuristic approaches which simply post-process real-valued solutions may not fully exploit the available target rank.

#### Scaling for larger number of patients:

In Table 5, we report the time spent for one iteration of increasingly larger subset of patients considered from the CMS data, with fixed target rank (*R* = 10). The time measured for the heuristic approaches corresponds to the execution time of CP-ALS, since the post-processing cost is negligible. We observe that SUSTain_*T*_ achieves linear scale-up w.r.t. increasing number of patients. We also remark that the dominant cost in both SUSTain_*T*_ and the CP-ALS is the MTTKRP computation, which explains the comparable running time.

### 4.4 Case study on Phenotyping HF patients

Cardiovascular disease (CVD) is the leading cause of death worldwide and heart failure (HF) is a dominant cause of morbidity and mortality. HF is traditionally characterized by reduced ejection fraction (HFrEF) and preserved ejection fraction (HFpEF). But, recent evidence suggests that HF is more heterogeneous than is reflected by ejection fraction. We used SUSTain to explore this heterogeneity in an incident HF cohort.

#### Cohort and data selection:

We select only the HF case patients from the Sutter PAMF dataset. For each incident HF case, we extracted data in the 12-months before and the 12-months after the initial HF diagnosis date, which resulted in 70, 531 clinical encounters. We used all the data modalities available, i.e., medication orders and indications and encounter diagnoses. The size of the resulting (patient-by-diagnosis-by-medication) tensor is 3, 497 × 396 × 367; the tensor contains a total of 92, 662 non-zero elements.

#### Choosing the number of phenotypes:

We use the stability-driven criterion introduced in [51]. The intuition behind this criterion is in promoting a target rank for which several runs with different initial points return reproducible factors. We choose the diagnosis factor matrix as the factor under assessment. Let ***D***_1_ and ***D***_2_ be the diagnosis factor matrix for 2 different runs with the same target rank. Then, the cross-correlation matrix $C\in {\mathbb{R}}^{R\times R}$ is computed between the columns of ***D***_1_, ***D***_2_ and the dissimilarity between them is computed as [51]:

$$\text{diss}\left(D_{1},D_{2}\right)=\frac{1}{2R}\left(2R-\overset{R}{\underset{j=1}{\sum }}{\text{max}}_{1\leq k\leq R}C(k,j)-\overset{R}{\underset{k=1}{\sum }}{\text{max}}_{1\leq j\leq R}C(k,j)\right)$$

Note that when ***D***_1_ can be transformed to ***D***_2_ by column permutation, then *diss*(***D***_1_, ***D***_2_) = 0. If *B* is the number of repetitions for each target rank, then the following relation computes the average dissimilarity over *B*(*B* − 1)/2 pairs of resulting factors:

$$Y(R)=\frac{2}{B(B-1)}\underset{1\leq b<b^{\prime }\leq B}{\sum }\text{diss}\left(D_{b},D_{b^{\prime }}\right)$$

We used the “staNMF” toolbox^3^ to compute the above score for each target rank on the range {5, … , 20}. The input to SUSTain_*T*_ were *B* = 20 initial points of the round heuristic. *R* = 15 phenotypes were selected based on the above criterion. For the target rank chosen, we pick the solution yielding the highest fit.

#### SUSTain provides concise and accurate solutions:

We observed that besides preserving the input data properties and providing a natural interpretation for medical experts, SUSTain implicitly imposes sparse factors. To assess the factors’ conciseness, we compare their fit with the achieved fit of the real-valued model (NN CP-ALS), which is post-processed to achieve factor sparsity (as would be done by a practitioner). For each of the feature factors (diagnosis and medication) of the real-valued model, we only consider the top-*k* elements for each column (i.e., most important elements of each phenotype). For the patient factor, we consider the top-*k* elements for each row (i.e., most important phenotypes for each patient). In each case, the value of *k* is chosen so that the sparsity level is close to the one achieved by SUSTain_*T*_ . We provide the results in Table 6, where we notice that for the same level of sparsity, SUSTain_*T*_ achieves ≈ 8.6% increase in fit. Thus, the integer factors of SUSTain_*T*_ decompose the input more accurately for the same level of sparsity than the real-valued counterpart.

#### Phenotype discovery:

In Table 1 and Table 7, we provide representative phenotypes extracted through our method. A subset of annotations provided by the cardiologist are as follows: hyperlipidemia (the one in Table 1), HF with reduced LVEF (HFrEF), hypertension (HTN), HTN which is more difficult to control, persistent and chronic atrial fibrillation, depression, diabetes, comorbidities of aging, prior pulmonary embolism. Overall, 13 out of 15 phenotype candidates were annotated as clinically meaningful phenotypes related to heart failure.

## 5 RELATED WORK

### Discrete factorization-based approaches:

Dong et al. [15] proposed an Integer Matrix Factorization framework via solving Integer Least Squares subproblems. As we experimentally evaluated, this approach is orders of magnitude slower than SUSTain while achieving the same level of accuracy. Kolda and O’Leary [30, 40] proposed a Semidiscrete Matrix Decomposition into factors containing ternary values ({−1, 0, 1}). Despite its demonstrated success for compression purposes, a direct application of this approach would introduce negative values into the factors, thus hurting interpretability for nonnegative input. Finally, several prior works target binary factorization (e.g., [32, 33, 36–38, 44, 53]). In contrast to strictly binary factors, SUSTain captures the quantity embedded in the input data, which reveals important information (e.g., relative phenotype prevalence and associated feature frequencies).

### Unsupervised Phenotyping:

Extensive prior work applies factorization techniques for unsupervised phenotyping (e.g., [19, 21, 22, 24, 41, 42, 50]). However, no work considered extracting scoring-based phenotypes to facilitate their interpretation by domain experts.

### HALS fitting algorithms:

Our fitting algorithms follow the intuition of Hierarchical Alternating Least Squares (HALS) framework [12] (aka rank-one residue iteration [23]), which enables formulating the solution for each *k*-th rank-1 component separately. However, plain HALS does not tackle the challenges involved with either imposing integer constraints or solving for the vector ***λ***.

## 6 CONCLUSIONS

The accuracy and scalability of SUSTain on “native” integer data derives from two key insights. One is expected: just rounding or applying related transformations to real-valued solutions is inherently limited. The second may be more surprising: while discrete constraints might appear to make the problem more challenging, in fact, a careful organization of the problem into subparts can mitigate that complexity. In our case, we identify a problem partitioning of integer-constrained subproblems that leads to an optimal and efficient solution; and, we also define the order of alternating updates so as to enable reuse of shared intermediate results. Consequently, SUSTain outperforms several baselines on both synthetic (publicly-available) and real EHR data, showing either a better fit or orders-of-magnitude speedups at a comparable fit.

Moving forward, there are many other sources of integer values in real-world data. These include, for instance, ordinal values. Thus, whereas this paper targets event counts, extensions for other cases is a ripe target for future work.

Lastly, to enable reproducibility of our work, we open-source our implementations and make them publicly available at: https://github.com/kperros/SUSTain.

## Figures and Tables

**Table 1:** Most prevalent phenotype (26% of patients) extracted via SUSTain_*T*_ for a heart failure cohort. The *r*-th phenotype prevalence is measured through the patient membership vectors containing non-zero element in the *r*-th coordinate. The score of each feature indicates its relative frequency. The prefix for each feature indicates whether it corresponds to a medication (Rx) or a diagnosis (Dx). The cardiologist labeled the result as “hyperlipidemia” and confirmed that the two features are clinically connected to heart failure.

Hyperlipidemia	Score
Rx_HMG CoA Reductase Inhibitors	3
Dx_Disorders of lipid metabolism	1

**Table 2:** Notations used throughout the paper.

Symbol	Definition
X, ***X***, ***x***, *x*	Tensor, matrix, vector, scalar
*vec*(***X***)	Vectorization operator for matrix ***X***
Π_*C*_ (***x***)	Euclidean projection of ***x*** to a set *C*
***X*** (:, *i*)	Spans the entire *i*-th column of ***X***
*diag*(***x***)	Diagonal matrix with vector ***x*** on the diagonal
***X***_(*n*)_	mode-***n*** matricization of tensor X
***A***^(*n*)^	factor matrix corresponding to mode ***n***
∘	Outer product
⊗	Kronecker product
⊙	Khatri-Rao product
$A_{⊙}^{\left(-n\right)}$	Khatri-Rao product of all the factor matrices expect ***A***^(*n*)^
***M***^(*n*)^	the MTTKRP corresponding to mode-***n***
*	Hadamard (element-wise) product

**Table 3:** For each dataset used, we list its name, nature of input modes, their sizes and the approximate number of non-zeros. Pat refers to patients, Dx to diagnoses, Rx to medications and Proc to procedures.

dataset	modes	size of modes	#nnz (≈Millions)
Sutter-matrix	Pat-Dx	259,999 × 576	5.7
Sutter-tensor	Pat-Dx-Rx	248,347 × 552 × 555	5.4
CMS-matrix	Pat-Dx	197,212 × 583	10.9
CMS-tensor	Pat-Dx-Proc	197,143 × 583 × 239	23.4

**Table 4:** Running time (seconds) of one iteration for increasingly larger number of patients considered from the CMS data. Matrix case, *R* = 10.

#patients (≈Thousands)	246	493	739	985
#nnz (≈Millions)	14	27	41	55
SUSTain_*M*_	0.71	0.95	1.66	2.82
Round / Scale-and-round	4.4	8.9	12.9	19.5
AILS	339	514	940	1254

**Table 5:** Running time (seconds) of one iteration for increasingly larger number of patients considered from the CMS data. Tensor case, *R* = 10.

#patients (≈Thousands)	246	493	739	985
#nnz (≈Millions)	29	58	88	117
SUSTain_*T*_	38.5	76.9	115	151
Round / Scale-and-round	39.6	78	117	157

**Table 6:** SUSTain_*T*_ achieves ≈ 8.6% increase in fit than a Nonnegative CP-ALS model truncated to achieve the same level of sparsity. The result refers to the HF case study for *R* = 15.

method	#nnz(A^(1)^)	#nnz(A^(2)^)	#nnz(A^(3)^)	fit
SUSTain_*T*_	3, 438	54	88	0.261
NN CP-ALS	3, 497	60	90	0.175

**Table 7:** Representative phenotypes extracted by SUSTain_*T*_ for our HF case study. The score of each feature indicates its relative frequency within the phenotype. The prefix for each feature indicates whether it corresponds to a medication (Rx) or a diagnosis (Dx). A cardiologist provided phenotype annotations and validated that: the top-most phenotype is aligned to guideline-based management of HF with reduced LVEF (HFrEF), the next one corresponds to typical hypertensive patients (common risk factor of HF) and the last one corresponds to hypertensive patients being more difficult to control.

HF with reduced LVEF (HFrEF)	Score
Rx_Loop Diuretics	3
Dx_Congestive heart failure	1
Rx_ACE Inhibitors	1
Rx_Alpha-Beta Blockers	1
Rx_Potassium	1
Hypertension	Score
Rx_ACE Inhibitors	3
Dx_Essential hypertension	1
Rx_Alpha-Beta Blockers	1
Rx_Beta Blockers Cardio-Selective	1
Rx_Calcium Channel Blockers	1
Rx_HMG CoA Reductase Inhibitors	1
Rx_Loop Diuretics	1
Rx_Thiazides and Thiazide-Like Diuretics	1
Hypertension (more difficult to control)	Score
Rx_Angiotensin II Receptor Antagonists	2
Rx_Beta Blockers Cardio-Selective	2
Rx_Calcium Channel Blockers	2
Dx_Essential hypertension	1
Rx_Antiadrenergic Antihypertensives	1
Rx_Loop Diuretics	1
Rx_Potassium	1

## CCS CONCEPTS

• Computing methodologies → Factorization methods; • Applied computing → Health informatics;

## Footnotes

1 In MDCalc, one can find a vast amount of such scores used in medicine.

2 These data can be downloaded from https://www.cms.gov/Research-Statistics-Data-and-Systems/Downloadable-Public-Use-Files/SynPUFs/DE_Syn_PUF.html

3 https://github.com/bdgp/staNMF

## References

[1] 2017. Clinical Classifications Software (CCS) for ICD-9-CM. https://www.hcup-us.ahrq.gov/toolssoftware/ccs/ccs.jsp. (2017). Accessed: 2017-02-11.

[2] Acar Evrim, Aykut-Bingol Canan, Bingol Haluk, Bro Rasmus, and Yener Bülent. 2007. Multiway analysis of epilepsy tensors. Bioinformatics 23, 13 (2007), i10–i18. 17646285 10.1093/bioinformatics/btm210

[3] Bader Brett W and Kolda Tamara G. 2007. Efficient MATLAB computations with sparse and factored tensors. SIAM Journal on Scientific Computing 30, 1 (2007), 205–231.

[4] Bader Brett W., Kolda Tamara G., and others. 2015. MATLAB Tensor Toolbox Version 2.6. Available online. (February 2015). http://www.sandia.gov/~tgkolda/TensorToolbox/

[5] Boyd Stephen and Vandenberghe Lieven. 2004. Convex optimization. Cambridge university press.

[6] Breen Stephen and Chang Xiao-Wen. 2012. Column Reordering for Box-Constrained Integer Least Squares Problems. (April 2012).

[7] Brewer John. 1978. Kronecker products and matrix calculus in system theory. IEEE Transactions on circuits and systems 25, 9 (1978), 772–781.

[8] Bro Rasmus and Sidiropoulos Nicholaos D. 1998. Least squares algorithms under unimodality and non-negativity constraints. Journal of Chemometrics 12, 4 (1998), 223–247.

[9] Carroll J Douglas and Chang Jih-Jie. 1970. Analysis of individual differences in multidimensional scaling via an N-way generalization of “Eckart-Young” decomposition. Psychometrika 35, 3 (1970), 283–319.

[10] Chang Xiao-Wen and Zhou Tianyang. 2007. MILES: MATLAB package for solving mixed integer least squares problems. GPS Solutions 11, 4 (2007), 289–294. Last updated: June 2016.

[11] Choi Edward, Schuetz Andy, Stewart Walter F, and Sun Jimeng. 2016. Using recurrent neural network models for early detection of heart failure onset. Journal of the American Medical Informatics Association 24, 2 (2016), 361–370. 10.1093/jamia/ocw112 PMC5391725 27521897

[12] Cichocki Andrzej and Phan Anh-Huy. 2009. Fast local algorithms for large scale nonnegative matrix and tensor factorizations. IEICE transactions on fundamentals of electronics, communications and computer sciences 92, 3 (2009), 708–721.

[13] Denny Joshua C, Ritchie Marylyn D, Basford Melissa A, Pulley Jill M, Bastarache Lisa, Brown-Gentry Kristin, Wang Deede, Masys Dan R, Roden Dan M, and Crawford Dana C. 2010. PheWAS: demonstrating the feasibility of a phenomewide scan to discover gene–disease associations. Bioinformatics 26, 9 (2010), 1205–1210. 20335276 10.1093/bioinformatics/btq126 PMC2859132

[14] Ding Chris, Li Tao, Peng Wei, and Park Haesun. 2006. Orthogonal nonnegative matrix t-factorizations for clustering. In Proceedings of the 12th ACM SIGKDD international conference on Knowledge discovery and data mining. ACM, 126–135.

[15] Dong Bo, Lin Matthew M, and Park Haesun. 2017. Integer Matrix Approximation and Data Mining. Journal of scientic computing (9. 2017), 1–27.

[16] Gillis Nicolas and others. 2011. Nonnegative matrix factorization: Complexity, algorithms and applications. Unpublished doctoral dissertation, Université catholique de Louvain. Louvain-La-Neuve: CORE (2011).

[17] Golub Gene H and Van Loan Charles F. 2013. Matrix Computations. Vol. 3. JHU Press.

[18] Harshman Richard A. 1970. Foundations of the PARAFAC procedure: Models and conditions for an “explanatory” multi-modal factor analysis. (1970).

[19] Henderson J, Ho JC, Kho AN, Denny JC, Malin BA, Sun J, and Ghosh J. 2017. Granite: Diversified, Sparse Tensor Factorization for Electronic Health Record-Based Phenotyping. In 2017 IEEE International Conference on Healthcare Informatics (ICHI). 214–223.

[20] Hitchcock Frank L. 1927. The expression of a tensor or a polyadic as a sum of products. Studies in Applied Mathematics 6, 1–4 (1927), 164–189.

[21] Ho Joyce C, Ghosh Joydeep, Steinhubl Steve R, Stewart Walter F, Denny Joshua C, Malin Bradley A, and Sun Jimeng. 2014. Limestone: high-throughput candidate phenotype generation via tensor factorization. Journal of biomedical informatics 52 (12. 2014), 199–211. 25038555 10.1016/j.jbi.2014.07.001 PMC6563906

[22] Ho Joyce C, Ghosh Joydeep, and Sun Jimeng. 2014. Marble: High-throughput Phenotyping from Electronic Health Records via Sparse Nonnegative Tensor Factorization. In Proceedings of the 20th ACM SIGKDD International Conference on Knowledge Discovery and Data Mining (KDD ‘14). ACM, New York, NY, USA, 115–124.

[23] Ho Ngoc-Diep. 2008. Nonnegative matrix factorization algorithms and applications. Ph.D. Dissertation. PhD thesis, Université catholique de Louvain.

[24] Joshi Shalmali, Gunasekar Suriya, Sontag David, and Joydeep Ghosh. 2016. Identifiable Phenotyping using Constrained Non-Negative Matrix Factorization. In Machine Learning for Healthcare Conference. 17–41.

[25] Kannan R, Ballard G, and Park H. 2018. MPI-FAUN: An MPI-Based Framework for Alternating-Updating Nonnegative Matrix Factorization. IEEE Transactions on Knowledge and Data Engineering 30, 3 (3 2018), 544–558. DOI:10.1109/TKDE.2017.2767592

[26] Karatzoglou Alexandros, Amatriain Xavier, Baltrunas Linas, and Oliver Nuria. 2010. Multiverse recommendation: n-dimensional tensor factorization for context-aware collaborative filtering. In Proceedings of the fourth ACM conference on Recommender systems. ACM, 79–86.

[27] Kim Jingu, He Yunlong, and Park Haesun. 2014. Algorithms for Nonnegative Matrix and Tensor Factorizations: A Unified View Based on Block Coordinate Descent Framework. Journal of Global Optimization 58, 2 (2. 2014), 285–319.

[28] Kim Jingu and Park Haesun. 2011. Fast nonnegative matrix factorization: An active-set-like method and comparisons. SIAM Journal on Scientific Computing 33, 6 (2011), 3261–3281.

[29] Kolda Tamara G and Bader Brett W. 2009. Tensor decompositions and applications. SIAM review 51, 3 (2009), 455–500.

[30] Kolda Tamara G and O’Leary Dianne P. 1998. A Semidiscrete Matrix Decomposition for Latent Semantic Indexing Information Retrieval. ACM Transactions on Information and System Security 16, 4 (10. 1998), 322–346.

[31] Kolda Tamara G and Sun Jimeng. 2008. Scalable tensor decompositions for multi-aspect data mining. In Data Mining, 2008. ICDM’08. Eighth IEEE International Conference on. IEEE, 363–372.

[32] Koyuturk Mehmet, Grama Ananth, and Ramakrishnan Naren. 2005. Compression, clustering, and pattern discovery in very high-dimensional discrete-attribute data sets. IEEE Transactions on Knowledge and Data Engineering 17, 4 (2005), 447–461.

[33] Koyutürk Mehmet, Grama Ananth, and Ramakrishnan Naren. 2006. Nonorthogonal decomposition of binary matrices for bounded-error data compression and analysis. ACM Transactions on Mathematical Software (TOMS) 32, 1 (2006), 33–69.

[34] Leader Joseph B, Pendergrass Sarah A, Verma Anurag, Carey David J, Hartzel Dustin N, Ritchie Marylyn D, and Kirchner H Lester. 2015. Contrasting association results between existing PheWAS phenotype definition methods and five validated electronic phenotypes. In AMIA Annual Symposium Proceedings, Vol. 2015. American Medical Informatics Association, 824. 26958218 PMC4765620

[35] Lee DD and Seung HS. 1999. Learning the parts of objects by non-negative matrix factorization. Nature 401, 6755 (10. 1999), 788–791. 10548103 10.1038/44565

[36] Lian Defu, Liu Rui, Ge Yong, Zheng Kai, Xie Xing, and Cao Longbing. 2017. Discrete Content-aware Matrix Factorization. In Proceedings of the 23rd ACM SIGKDD International Conference on Knowledge Discovery and Data Mining. ACM, 325–334.

[37] Miettinen Pauli. 2011. Boolean tensor factorizations. In Data Mining (ICDM), 2011 IEEE 11th International Conference on. IEEE, 447–456.

[38] Miettinen P, Mielikäinen T, Gionis A, Das G, and Mannila H. 2008. The Discrete Basis Problem. IEEE transactions on knowledge and data engineering 20, 10 (10. 2008), 1348–1362.

[39] Nahler Gerhard. 2009. anatomical therapeutic chemical classification system (ATC). Springer Vienna, Vienna, 8–8. DOI:10.1007/978-3-211-89836-9_64

[40] O’Leary D and Peleg S. 1983. Digital Image Compression by Outer Product Expansion. IEEE Transactions on Communications 31, 3 (3 1983), 441–444.

[41] Perros Ioakeim, Chen Robert, Vuduc Richard, and Sun Jimeng. 2015. Sparse hierarchical tucker factorization and its application to healthcare. In Data Mining (ICDM), 2015 IEEE International Conference on. IEEE, 943–948.

[42] Perros Ioakeim, Papalexakis Evangelos E, Wang Fei, Vuduc Richard, Searles Elizabeth, Thompson Michael, and Sun Jimeng. 2017. SPARTan: Scalable PARAFAC2 for Large & Sparse Data. In Proceedings of the 23rd ACM SIGKDD International Conference on Knowledge Discovery and Data Mining (KDD ‘17). ACM, 375–384.

[43] Richesson Rachel L, Sun Jimeng, Pathak Jyotishman, Kho Abel N, and Denny Joshua C. 2016. Clinical phenotyping in selected national networks: demonstrating the need for high-throughput, portable, and computational methods. Artificial intelligence in medicine 71 (7 2016), 57–61. 27506131 10.1016/j.artmed.2016.05.005 PMC5480212

[44] Shen Bao-Hong, Ji Shuiwang, and Ye Jieping. 2009. Mining Discrete Patterns via Binary Matrix Factorization. In Proceedings of the 15th ACM SIGKDD International Conference on Knowledge Discovery and Data Mining (KDD ‘09). ACM, New York, NY, USA, 757–766.

[45] Slee Vergil N. 1978. The International classification of diseases: ninth revision (ICD-9). Annals of internal medicine 88, 3 (1978), 424–426. 629506 10.7326/0003-4819-88-3-424

[46] Smilde Age, Bro Rasmus, and Geladi Paul. 2005. Multi-way analysis: applications in the chemical sciences. John Wiley & Sons.

[47] Takapoui Reza, Moehle Nicholas, Boyd Stephen, and Bemporad Alberto. 2017. A simple effective heuristic for embedded mixed-integer quadratic programming. Internat. J. Control (2017), 1–11.

[48] Ustun Berk and Rudin Cynthia. 2017. Optimized Risk Scores. In Proceedings of the 23rd ACM SIGKDD International Conference on Knowledge Discovery and Data Mining. ACM, 1125–1134.

[49] Chang X w. and Han Q. 2008. Solving Box-Constrained Integer Least Squares Problems. IEEE Transactions on Wireless Communications 7, 1 (1. 2008), 277–287.

[50] Wang Yichen, Chen Robert, Ghosh Joydeep, Denny Joshua C, Kho Abel, Chen You, Malin Bradley A, and Sun Jimeng. 2015. Rubik: Knowledge Guided Tensor Factorization and Completion for Health Data Analytics. In Proceedings of the 21th ACM SIGKDD International Conference on Knowledge Discovery and Data Mining (KDD ‘15). ACM, New York, NY, USA, 1265–1274. 10.1145/2783258.2783395 PMC6709413 31452969

[51] Wu Siqi, Joseph Antony, Hammonds Ann S, Celniker Susan E, Yu Bin, and Frise Erwin. 2016. Stability-driven nonnegative matrix factorization to interpret spatial gene expression and build local gene networks. Proceedings of the National Academy of Sciences 113, 16 (2016), 4290–4295. 10.1073/pnas.1521171113 PMC4843452 27071099

[52] Yadav Pranjul, Steinbach Michael, Kumar Vipin, and Simon Gyorgy. 2018. Mining Electronic Health Records (EHRs): A Survey. ACM Computing Surveys (CSUR) 50, 6 (2018), 85.

[53] Zhang Z, Li T, Ding C, and Zhang X. 2007. Binary Matrix Factorization with Applications. In Seventh IEEE International Conference on Data Mining (ICDM 2007). 391–400.

